# Multimodal epidermal devices for hydration monitoring

**DOI:** 10.1038/micronano.2017.14

**Published:** 2017-06-05

**Authors:** Siddharth Krishnan, Yunzhou Shi, R. Chad Webb, Yinji Ma, Philippe Bastien, Kaitlyn E. Crawford, Ao Wang, Xue Feng, Megan Manco, Jonas Kurniawan, Edward Tir, Yonggang Huang, Guive Balooch, Rafal M. Pielak, John A. Rogers

**Affiliations:** 1 Department of Materials Science and Engineering, Frederick Seitz Materials Research Laboratory, University of Illinois at Urbana-Champaign, Urbana, IL 61801, USA; 2 Department of Materials Science and Engineering, Northwestern University, Evanston, IL 60208, USA; 3 L’Oreal Tech Incubator, California Research Center, 953 Indiana Street, San Francisco, CA 94107, USA; 4 Department of Civil and Environmental Engineering, Mechanical Engineering, Materials Science and Engineering, Northwestern University, Evanston, IL 60208, USA; 5 L’Oréal Research and Innovation, 1 Avenue Eugène Schuller, Aulnay sous Bois 93601, France; 6 Department of Engineering Mechanics, Center for Mechanics and Materials, Tsinghua University, Beijing 100084, China; 7 L’Oréal Early Clinical, 133 Terminal Avenue, Clark, NJ 07066, USA; 8 Departments of Materials Science and Engineering, Biomedical Engineering, Chemistry, Mechanical Engineering, Electrical Engineering and Computer Science, and Neurological Surgery; Center for Bio-Integrated Electronics; Simpson Querrey Institute for Nano/biotechnology; Northwestern University, Evanston, IL 60208, USA

**Keywords:** epidermal electronics, hydration monitoring, thermal characterization

## Abstract

Precise, quantitative *in vivo* monitoring of hydration levels in the near surface regions of the skin can be useful in preventing skin-based pathologies, and regulating external appearance. Here we introduce multimodal sensors with important capabilities in this context, rendered in soft, ultrathin, ‘skin-like’ formats with numerous advantages over alternative technologies, including the ability to establish intimate, conformal contact without applied pressure, and to provide spatiotemporally resolved data on both electrical and thermal transport properties from sensitive regions of the skin. Systematic *in vitro* studies and computational models establish the underlying measurement principles and associated approaches for determination of temperature, thermal conductivity, thermal diffusivity, volumetric heat capacity, and electrical impedance using simple analysis algorithms. Clinical studies on 20 patients subjected to a variety of external stimuli validate the device operation and allow quantitative comparisons of measurement capabilities to those of existing state-of-the-art tools.

## Introduction

Skin, the largest organ of the human body, is critical to many physiological processes. The skin also serves as a diffusion barrier^1^, both to block penetration of undesired pathogens^2^, and to prevent excessive transepidermal water loss^3^. The outermost part of skin, the stratum corneum, and the immediate underlying tissue, the epidermis, consist of layered collections of avascular, keratinized cells, in an extracellular lipid matrix^4,5,6,7^. Structurally, unbound, ‘free’ water that easily diffuses through skin layers, and molecularly ‘bound’ water that is largely confined to its bonding sites^8^ in the stratum corneum are essential to its barrier function. Improper hydration can lead to eczema and accelerated aging of the skin, and to adverse effects on appearance.

Traditional sensors of skin hydration rely on capacitive or impedance-based methodologies in which mechanical force establishes contact to rigid measurement electrodes^9,10^. The most commonly used tool for making such measurements, the corneometer (Courage+Khazaka electronic GmbH), relies on capacitive sensing based on concentric ring electrodes (circular inner electrode ~5 mm diameter, annular outer electrode ~9 mm inner diameter, ~22 mm outer diameter^10^), with typical operating frequencies in the MHz range. The applied pressure that is necessary to form the electrical interface with such an instrument also compresses and deforms the skin, thereby altering its hydration characteristics and capacitive properties. As a result, reliable measurements demand the use of precisely calibrated contact pressures. Even with such approaches, reproducibility can be a challenge^10^. Other important limitations include an inability to evaluate sensitive parts of the body, such as the face, and to allow continuous monitoring, either in a clinic or a home setting.

Here we describe recent advances in multimodal sensors of skin hydration, via measurements of intrinsic thermal and electrical properties, in which advanced materials, mechanics and concepts render devices that have soft, ‘skin-like’, or ‘epidermal’, formats^11,12,13,14,15,16,17,18,19,20^. The thin geometries and compliant mechanics lead to a mode of integration with the skin that does not require application of pressure, and instead relies on van der Waals adhesion forces alone. The device and measurement interface are mechanically imperceptible to the user, and therefore compatible for evaluation on any part of the body, in a manner that obviates the need for pressure sensing and offers potential for use in continuous, long-term recordings. The results outlined in the following extend our previously reported platforms for this purpose by (1) integrating both thermal and electrical characterization capabilities into a single measurement system, (2) introducing simple algorithms for extracting a full complement of intrinsic thermal and electrical properties from the measurement data, with good quantitative correspondence to simple models of the skin, and (3) applying the technology in a comprehensive clinical trial, with 20 patients and a variety of skin stimuli, for validation and comparison against clinical standard tools, that is, corneometers. The findings establish the design considerations, the analysis approaches and the capabilities of this technology for use in scenarios ranging from bedside hospital care, to clinical evaluation and study, to at-home diagnostics.

The thermal^21,22^ and electrical properties^23^ of skin both depend strongly on water content, but for different reasons. Consequently, devices that offer, in a single platform, capabilities for measuring both the thermal and the electrical characteristics of the skin provide improved insights into the state of hydration. In general, thermal transport techniques probe^15,24^ the flow of heat deep into the epidermis. Here, the response changes with hydration simply because the thermal properties of water are different than those of the tissue itself. By contrast, measurements of electrical impedance involve currents that can localize primarily to the stratum corneum^25^, where the high frequency dielectric characteristics of free and bound water affect the results in a way that correlates to overall hydration level. In both cases, various key parameters such as device geometry, measurement power and frequency, determine the characteristics of the measurement, including the depth sensitivity.

**Figure 1:**
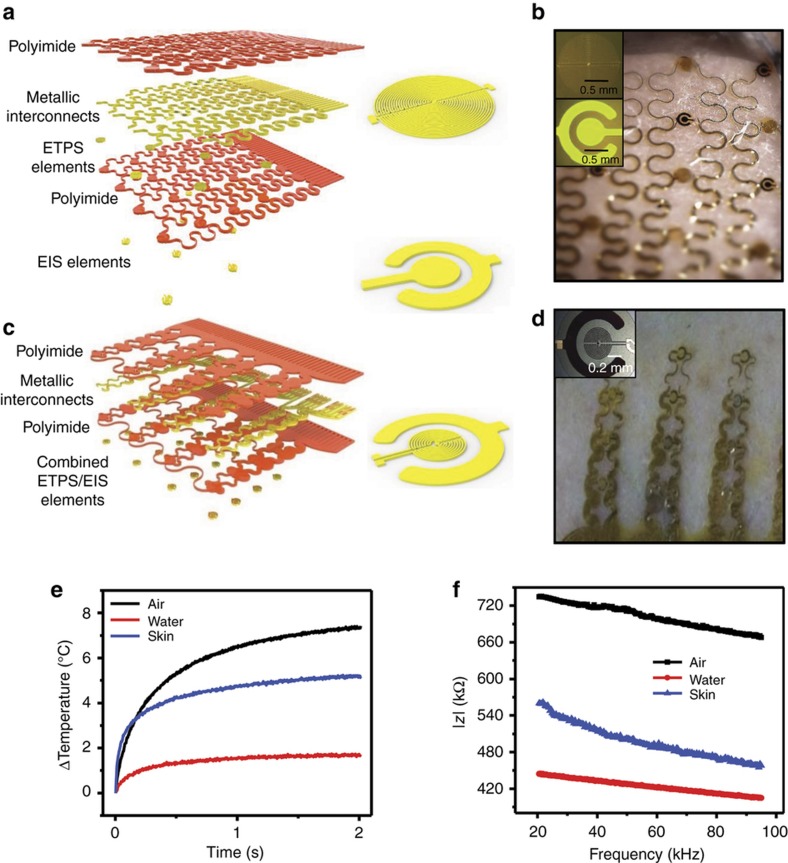
Multimodal electronics for epidermal hydration mapping. (**a**) Exploded view schematic of device showing different layers (inset: enlarged view of epidermal transient plane source (ETPS) sensor (top) and epidermal impedance sensor (EIS, bottom). (**b**) Optical image of sensor with separate ETPS and EIS elements integrated onto the same device. (inset) Optical micrographs of EIS (top) and ETPS sensor (bottom). (**c**) Exploded view schematic of device with combined ETPS and EIS elements (inset: enlarged view of combined sensing element). (**d**) Optical image of device with combined EIS and ETPS elements (inset: optical micrograph of combined ETPS/EIS sensor). (**e**) Transient thermal data recorded for three materials with different thermal properties, from combined sensor shown in **c** and **d**. (**f**) Impedance spectra shown for three materials with different electrical properties from combined sensor shown in **c** and **d**.

The results presented here build on our previously reported epidermal devices for thermal and electrical measurements of the skin. The advances are in device configurations, approaches for data analysis, effective medium models for hydrated skin, and parameter extraction and clinical validation. Thermal and electrical modalities exist in a single platform, as in Figures 1a–d, where a collection of individual sensing elements function as thermal sensors/actuators and as impedance electrodes. Switching between the two modalities occurs by simple multiplexed control by the user for small arrays, or by a microcontroller for large arrays. Operation involves external electronics and wired connections.

The geometries of the impedance electrodes, which we refer to as epidermal impedance sensors (EIS), and their optimization for hydration measurement appear elsewhere^26^. The thermal sensors, which we refer to here as epidermal transient plane source (ETPS) sensors, with appropriate data analysis algorithms, can simultaneously determine thermal conductivity (*k*) and diffusivity (*α*), and, through the ratio of these two quantities, the volumetric heat capacity (*ρC*_p_). Systematic *in vitro* studies, including comparisons to results of differential scanning calorimetry (DSC) and separate literature reports for a range of materials validate the measurement capabilities and the analysis approach. Finite element models establish measurement depths for both thermal and electrical modalities, for a range of skin hydration levels. A trial involving 20 patients provides statistically meaningful evidence for the suitability of these device platforms for use in a hospital or clinical settings, supported by comparisons to existing state-of-the-art reference measurements.

## Materials and methods

### Fabrication of integrated sensors

Figure 1a shows an exploded view schematic illustration of the device. The thermal sensors consist of resistive elements that serve as thermal actuators, via Joule heating, and simultaneously as temperature sensors, via the coefficient of resistance (TCR) of the metal. The EIS elements consist of concentric ring electrodes capable of injecting alternating current into the skin, through the physical contact interface. Thin layers of polyimide insulate all of the metal structures in the ETPS components, and the interconnect wiring for the EIS elements. Photolithography defines the geometries with submicron precision. Open mesh layouts, combined with thin elastomer substrates (50 μm silicone; Ecoflex, Smooth-On Inc., Macungie, PA, USA) yield soft, ‘epidermal’ mechanics, based on design principles well established by the stretchable electronics community^12,27–32^. Connections to external data acquisition and powering electronics use thin ribbon wiring based on anisotropically conducting film (ACF; Elform, Reno, Nevada, USA), thermally bonded to exposed pads located at the periphery of the device.

Fabrication and design details appear in the SI. Briefly, the processing involves first spin-casting and curing a sacrificial layer of (poly)methyl-methacrylate (PMMA, 500 nm thick) (Microchem, Westborough, MA, USA) onto a clean 300 mm silicon wafer. A film of polyimide (PI 2545, Parlin, NJ, USA, 1.2 μm thick) spin-cast and cured on top of this layer forms the bottom side of the encapsulating film. The ETPS and EIS, respectively, layers consist of thin-film, metallic elements. Both sets of sensing elements use a bilayer of Cr (10 nm)/Au (100 nm) deposited by electron beam evaporation and patterned by photolithography. A multilayer of Ti (20 nm)/Cu (550 nm)/Ti (20 nm)/Au (25 nm), deposited and patterned in a similar manner, defines the interconnect layer in an open mesh serpentine layout that provides stretchable mechanics when mounted on an elastomer substrate. A second layer of PI layer forms the top encapsulating film. Immersing the wafer in warm acetone dissolves the PMMA, thereby releasing the devices. Retrieval using a Polyvinyl Alcohol (PVA)-based water soluble tape (3 M, Minneapolis, MN) followed by deposition of a thin layer of SiO_2_ facilitates adhesive bonding to a thin (50 μm) silicone-based substrate (Ecoflex, Smooth-On Inc., Macungie, PA, USA). Removing the PVA by immersion in warm water completes the fabrication.

### *In vitro* studies of hydration

**Figure 2:**
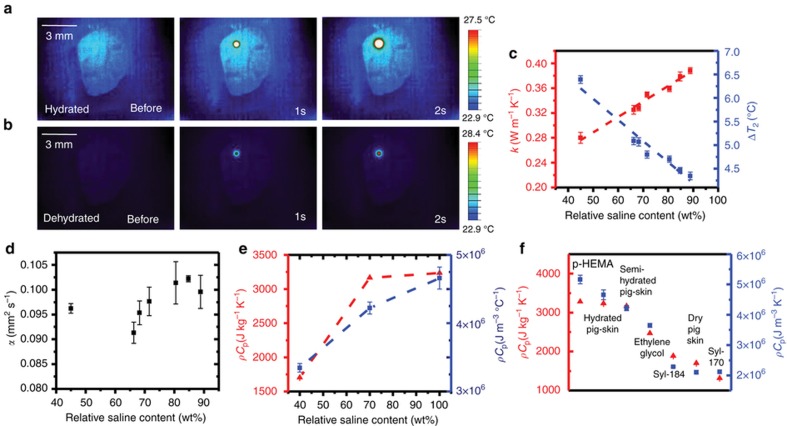
ETPS sensors of epidermal hydration and volumetric heat capacity. (**a**) Infrared (IR) image of epidermal 1 mm epidermal transient plane source (ETPS) element on hydrated pig skin (90% saline by weight) before heating, 1 and 2 s after heating starts, respectively. (**b**) IR images of same sensor, at same time points, on dehydrated pig skin (45% saline by weight). (**c**) Thermal conductivity (red) and maximum temperature attained at 2 s (blue) as measured by 1 mm ETPS sensor on pig skin at different hydration levels. (**d**) Thermal diffusivity measured by ETPS on pig skin sample at different hydration levels. (**e**) Volumetric heat capacity derived from ETPS sensor (blue) and specific heat capacity measured by differential scanning calorimetry (DSC, red), on pig skin samples at different hydration levels. (**f**) Volumetric heat capacity (blue) measured by ETPS sensor, plotted against specific heat capacity measured by DSC for a range of test materials with properties similar to those of skin. In **c**–**f**, error bars represent standard deviation across four elements, each measured three times.

Studies involved porcine skin from the stomach region, acquired in frozen form. Thawing the tissue at room temperature for ~12 h and paring away the underlying fat layers using a scraping knife prepared samples (25×25×5 mm) for testing. Soaking in a bath of phosphate-buffered saline (pH=7.4) led to gradual uptake of water. The epidermal sensors and an analytical balance allowed measurements of thermal characteristics and the mass, respectively, of samples removed from the bath at several time points. Each evaluation involved three measurements from three separate sensors, for a total of nine measurements. The error bars in Figures 2c–f represent the full range of these data. The process of fitting the data to determine the thermal conductivity and diffusivity involved a custom algorithm (MATLAB, Mathworks, Natick, Massachusetts, USA) based on the solution to the heat diffusion equation for a transient plane source.

### Simulation of thermal and electrical fields

#### Finite element analysis

Computational studies of the thermal and electrical responses relied on commercial software from ABAQUS^9^ and COMSOL^10^, respectively. For the former, the encapsulation layer (Ecoflex), skin, and heater used axisymmetric heat transfer elements (DCAX4). For the latter, the encapsulation layer (Ecoflex), skin, and capacitor used axisymmetric electrical current elements.

### Clinical protocol

The cohort of patients consisted of 20 healthy, female volunteers recruited by Stephens & Associates (Dallas, Texas, USA). Approval by Stephens & Associates, IRB: Protocol No. C15-D088 (ACR-THERM-1515), with skin photo type II–IV according to the Fitzpatrick scale and intact, healthy skin in the forearm region. Two groups involved individuals with ages of 18–30 and 50–60 years. Four demarcated locations, each 25×25 mm on the volar forearm, labeled A, B, C, D served as the focus of the measurements. Location A involved an occlusive patch applied for 30 min, to prevent transdermal water loss. Locations B, C, and D, involved 20 mg of moisturizing lotions with glycerine concentrations of 0, 15, and 30%, respectively, applied in a randomized manner. The treatments followed a double-blind procedure. Data consisted of recordings before, immediately after, and at 30, 60, and 270 min after application of the compound. A final measurement (300 min after initial application) on these same regions followed immediately after thorough cleaning and drying with an absorbent pad. At each measurement time, the epidermal devices yielded thermal and impedance data. A corneometer probe (Cutometer MPA 580; Courage+Khazaka GmbH, Köln, Germany) yielded hydration levels, captured in three sequential measurements. Additional standard sensors (Tewameter TM 300; Courage+Khazaka GmbH) provided data on transepidermal water loss. Thermal transport data included transient temperature responses from 14 individual thermal actuator/sensor elements, each sequentially activated with input power of 7 mW mm^−2^ for 2 s using custom data acquisition electronics. Descriptions of curve fitting procedures appear in Supplementary Note S2. Impedance data corresponded to measurements with an AC voltage applied to the concentric ring electrodes (2 V, peak-to-peak) at frequencies between 10 kHz to 1.2 MHz, using a microcontroller (AD 5988 EBZ, Analog Instruments).

### Differential scanning calorimetry for specific heat capacity

Under a constant heating rate, *β*, in a DSC instrument, the deviation of the temperature of the sample relative to a reference *h,* defines the specific heat capacity, *C*_p_, according to *C*_p_=*h*/(*Bβ*) (Ref. 33), where *B* is a calibration factor determined from analysis of an established test material. The materials measured in Figures 2d and e were inserted into hermetically sealed 25 mg Aluminum pans in a DSC instrument (TA Instruments, Q100), and heated at a rate of 5 °C min^−1^ from room temperature to 150 °C. Measurements on sapphire determined the value of *B*. Representative DSC scans appear in Supplementary Figure S1.

### Statistical analysis

Scatterplot matrices of the descriptors as a function of time reveal pairwise relationships (Supplementary Figure S2). Box plot representations (Supplementary Figure S3) illustrate, for the different descriptors, raw responses and changes associated with treatments using creams with various glycerin content. Values corresponding to areas under the curves (AUC; Supplementary Figure S4) summarize individual profiles, and appear for different glycerin content using density plots. All statistical analyses used SAS software release 9.3, SAS Institute Inc., Cary, NC, USA, and JMP statistical software release 10.0 (JMP is a trademark of SAS Institute).

## Results and discussion

### Epidermal thermal transport properties and their relationship with hydration

The transient, spatially averaged change in temperature across an ETPS element with circular geometry on a semi-infinite medium, when effects of convection and the detailed multilayer construction of the device are ignored, can be written^24^ as:

(1)ΔT(τ)¯=P0(π32ak)−1D(τ)

where *P*_0_ is the total applied power, *a* is the radius of the element, *k* is the thermal conductivity of the semi-infinite medium and *D*(*τ*) is given by

(2)D(τ)=∫0τdσσ2∫01udu∫01vdv×exp(−(u2+v2)4σ2)I0(uv2σ2)

where *τ* is a dimensionless measure of time according to

(3)τ=tα/a2

where *α* is the thermal diffusivity of the semi-infinite medium, *t* is time and *I*_0_ is the second order Bessel function. In this way, both the thermal conductivity and the diffusivity influence the transient thermal response of each ETPS element. The characteristic diffusion length is^24^:

(4)Λ=Παt

where *Π* is a dimensionless factor on the order of unity. For a typical measurement time of 2 s, and a thermal diffusivity of 0.1 mm^2^ s^−1^, *Λ* is ~450 μm. Because the total thickness of the device is 50 μm, these results suggests that the individual layers in the overall construction can be treated as a single, effective thermal medium. Calibration using materials with known values of *k* (water, ethylene glycol) determines an effective measurement radius, *a*, that accounts for simplifications associated with assumptions used in this model. The radius determined in this manner is ~254 μm, approximately half of the true radius of the sensor (500 μm), is consistent with previous versions of related sensors^34^. *D*(*τ*), computed for a relevant range of *τ*, and a representative experimental data set with its corresponding fit appear in Supplementary Figure S5.

For ease of processing large quantities of data such as those obtained in clinical trials described subsequently, a simplified analytical expression^15^ can be useful,

(5)T=T∞+A1P02πA2kerfc(A2α2)

where *A*_1_ and *A*_2_ are constants selected to account for the layered device geometry, the effect of device edges, convective heat transport, and the distribution of temperature across the area of the element. The values correspond to those that yield the best match between experimental measurements on materials with known thermal properties similar to skin (water, ethylene glycol) and simulations based on Ref. 4. The approximately linear dependence of *k* on water content^35^ makes this quantity useful in determining hydration levels, as examined in detail below.

As an illustration of the thermal physics, infrared (IR) thermography results in Figures 2a and b reveal the distributions of temperature associated with operation of an isolated thermal actuator on hydrated (90% saline solution by weight) and dried (30% saline solution by weight) porcine skin, respectively. The hydration level clearly has a strong effect on the thermal behaviors. Water in the outer layers of the epidermis can broadly be divided into tightly bound ‘primary’ water, less tightly bound ‘secondary’ water, and completely unbound ‘free’ water^8^. Secondary water, as the main source of water in the epidermis (typically around 35 mg/100 mg of dry skin), most strongly influences its thermal properties, as well as its electrical conductivity and dielectric permittivity. An increase in secondary water content increases *k*, and thereby reduces the temperature throughout the duration of the measurement, and decreases the temperature gradients across and beyond the boundaries of the device. We note that although we did not observe any significant dependence of the adhesion strength on skin hydration state, specialized substrates can be useful to maintain adhesion during vigorous sweating^11^.

Values of *k* obtained by fitting data collected from a porcine skin sample using Equation (1), across a range of relative hydration levels, are in Figure 2c (red). Here 100% corresponds to the fully wet state, defined as the point at which further soaking does not change the weight of the sample. The error bars correspond to the full range of variations observed across four different sensors at different locations of a single sample, and also across three measurements performed with each of these sensors. Location-to-location variations are much larger than those associated with repeated measurements at a single location with a single sensor. Overall, increases in *k* correlate to decreases in the temperature rise, as expected intuitively. The temperatures recorded at each element 2 s after thermal actuation appear in Figure 2c (blue), with error bars computed the same way as above.

Figure 2d shows the thermal diffusivity, *α*, extracted in a similar manner. The error bars are larger, in a fractional sense, for *α* than for *k*, as a result of the relatively low sensitivity of the thermal response to the former. Thermal diffusivity does not vary in a linear manner with hydration because of its dual-dependence on *k* and *ρC*_p_. For hydration levels across a range that corresponds to healthy, living tissue, *α* varies by only a modest amount. Extreme decreases in hydration can, however, cause large decreases in this parameter.

Values for *ρC*_p_ determined from *k/α* for three distinct hydration regimes, are in Figure 2e. Comparisons against values obtained by DSC indicate good agreement. The relatively low variation of *ρC*_p_ with hydration level (typically ~20%, between ~900 kg m^−3^ for dry skin and 1100 kg m^−3^ for hydrated skin) allows variations in *ρC*_p_ to be equated with those in *C*_p_. Further studies involving a range of test materials, as shown in Figure 2f, suggest the utility of the ETPS sensor as a type of skin-integrated DSC for *in vivo* measurements. Transient temperature rise curves corresponding to the ETPS data shown in Figure 2f appear in Supplementary Figure S1c.

**Figure 3:**
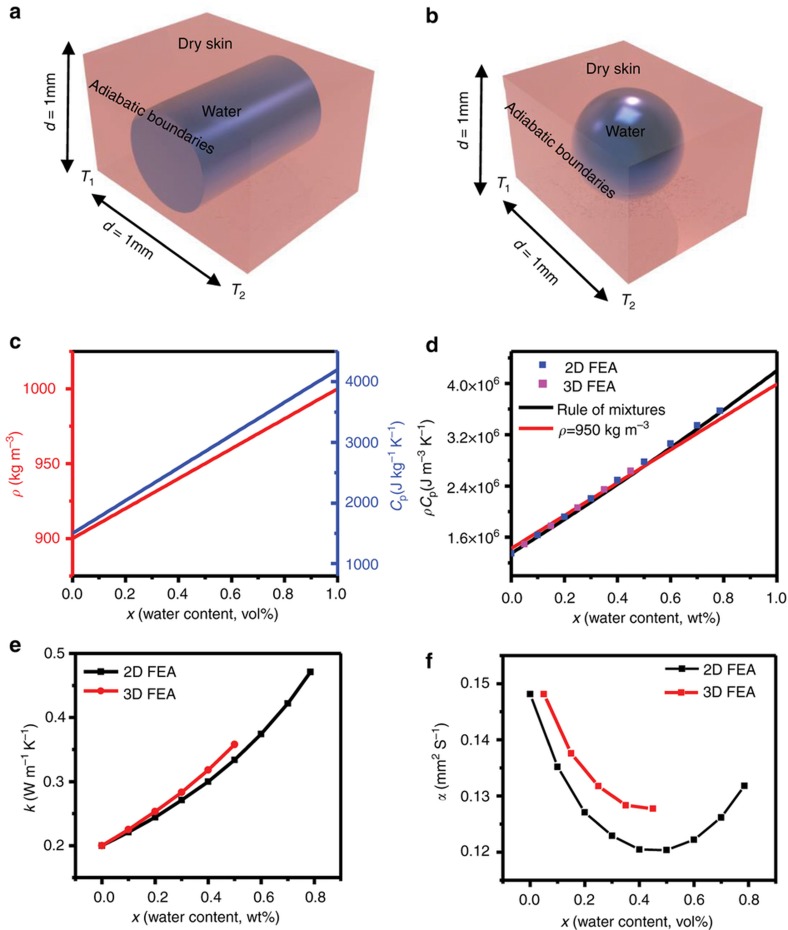
Effective medium model to predict trends in thermal properties with hydration. (**a**) Schematic illustrating single, two-dimensional (2D) cell used for finite element analysis (FEA) analysis for simulating changes in thermal properties with skin hydration. (**b**) Schematic illustration of single three-dimensional (3D) cell for simulating changes in thermal properties with skin hydration. (**c**) Density (red line) and specific heat capacity (blue line) computed as a function of water content using the rule of mixtures. (**d**) Simulated changes in volumetric heat capacity with hydration using 2D FEA (blue points), 3D FEA (pink points), the rule of mixtures (black line), and the rule of mixtures assuming a constant density (red line). (**e**) Simulated changes in thermal conductivity with water content computed via 2D FEA (black curve) and 3D FEA (red curve). (**f**) Simulated changes in thermal diffusivity with water content computed via 2D FEA (black curve) and 3D FEA (red curve).

The volume of skin under experimental investigation includes the hardened, keratinized stratum corneum and underlying layers of squamified epithelial cells that make up the majority of the epidermis. These different types of cells have different densities, heat capacities, and thermal conductivities, with gradual transitions between layers. To gain some insight into some of the trends in *ρC*_p_, *k*, and *α* with hydration, this complex structure can be approximated using simple effective medium models^36^ in which the epidermis with secondary water corresponds to a periodic array of square cells of side length *d*, each of which consists of a matrix of dry skin with a certain volume fraction of water, *x*_cell_ (0<*x*_cell_<1, where 0 is entirely dry skin, 1 is pure water). Two standard structures (Figures 3a and b), well established by previous reports^36^, can be considered. The first structure models water as a cylinder inside a square matrix of dry skin (Figure 3a), with a maximum geometrically allowed fill factor of 78%. The translational symmetry of this system in the direction parallel to the cylinder allows for a simple 2D approximation. The second structure models water as a spherical droplet inside a square matrix of dry skin (Figure 3b), with a maximum geometrically allowed fill factor of 50%, and requires a full 3D treatment. This range encompasses that of secondary water that naturally occurs in the outer epidermis (approximately 35–50% of the outer epidermis by weight), as reported elsewhere^8^. For computations, *d*=1 mm, and periodic repetition of this unit cell approximates the bulk, effective material. For heat, *Q*_cell_, introduced into this cell, with adiabatic boundaries, (*ρC*_p_)_cell_ can be written:

(6)(ρCp)cell=QcellΔTcellVcell

where *V*_cell_ is the volume of the cell and Δ*T*_cell_ is the steady-state change in temperature. Finite element analysis (FEA) can capture values of Δ*T*_cell_ as a function of *x*_cell_, in both the 2D and 3D cases outlined above. These results, along with Equation (6), define the dependence of *ρC*_p_ on *x*, which agrees with a simple rule of mixtures, (*ρ* and *C*_p_ are computed using the rule of mixtures appear in Figure 3c), in both the 2D and 3D cases for each quantity, as shown in Figure 3d. Here, the values of *C*_p_ for water and the dry skin matrix are 4200 and 1500 J kg^−1^ K^−1^, respectively, and the corresponding densities are 1000 and 900 kg m^−3^. As expected, *ρC*_p_, for the 2D and 3D models are the same.

To calculate *k*_cell_, we consider opposite edges of the cell fixed at *T*_1_=0 K and *T*_2_=1 K. For a heat flux of ${\dot{Q}}_{\mathrm{cell}}$ (where $\dot{Q}=\frac{1}{A}\;\frac{dQ}{dt}$, *t*=time) across this cell, *k*_cell_ can be stated by rearranging Fourier’s law to yield:

(7)kcell=Q˙celldcell(T2−T1)

FEA results yield values of ${\dot{Q}}_{\mathrm{cell}}$ for each value of *x*_cell_, thereby allowing calculation of the dependence of *k*_cell_ on *x*_cell_ via Equation (7). The results, which appear in Figure 3e for the 2D (black curve) and 3D cases (red curve), indicate a slightly non-linear dependence of *k* with *x* for the full range of *x*, but which can be treated with a linear approximation for narrower ranges of *x*_cell_ (for example, 0<*x*_cell_<50%), relevant to physiological conditions. Analytical variations of *k*_cell_ with *x*_cell_ using a rule of mixtures, for different geometries, appear in Supplementary Figure S6. Two extreme cases, modeled by the rule of mixtures and inverse rule of mixtures, bound these numerical results, as an independent validation.

The ratio of *k* to *ρC*_p_ defines the thermal diffusivity, *α*. The functional dependence of *α* on *x*, determined in this manner, appears in Figure 3f, for both the 2D and 3D cases. The slope, d*α/*d*x*, results from contributions from the functional dependence of the product of two factors, *k* and 1/(*ρC*_p_), as:

(8)α(x)=k(x)ρCp(x)

Using the rule of mixtures approximation, *ρ*(x) can be written as

(9)ρ(x)=900+100x

where 900 kg m^−3^ is the density of dry skin and 1000 kg m^−3^ is the density of water. Similarly, to a very good approximation^37^, *C*_p_(*x*) can be written as

(10)Cp(x)=1500+2700x

where 1500 J kg^−1^ K^−1^ is *C*_p_ for dry skin and 4200 J kg ^−1^ K^−1^ is *C*_p_ for water. Graphical representations of these functional forms appear in Figure 3c. Taking the derivative of the combined quantity, *ρC*_p_, with respect to *x* yields:

(11)dρCpdx=100(1500+2700x)+2700(900+100x)

where the first and second terms on the right represent contributions from changes in *ρ* $\left(\frac{d\rho }{dx}=100\right)$, and *C*_p_ $\left(\frac{dC_{p}}{dx}=2700\right)$, respectively. In the limit where *x*=0,

(12)dρCpdx=100(1500)+2700(900)=(1.5×104)+(2.43×106)

Similarly, in the limit *x*=1,

(13)dρCpdx=100(4200)+2700(1000)=(4.2×105)+(2.7×106)

In both of these extremes, changes in *ρC*_P_ are dominated by changes in *C*_p_, by two and one order of magnitude in Equations (12) and (13), respectively. A reasonable approximately, then, is that $\frac{d\rho C_{p}}{dx}\approx \bar{\rho }\frac{dC_{p}}{dx}$, where $\bar{\rho }$ is a constant, averaged value. A representative case, in which $\bar{\rho }$ is constant at 950 kg m^−3^ appears in Figure 3d (red curve), closely follows the FEA-computed curves (blue and pink points) and the rule-of-mixtures curve (black curve) in Figure 3d. Taking the derivative of Equation (8) yields:

(14)dα(x)dx=ρ¯Cp(x)dk(x)dx−k(x)ρ¯dCp(x)dx[ρ¯Cp(x)]2

This expression indicates that, unlike *k*, *α* does not necessarily vary in a monotonic fashion with hydration. Specifically, for the case considered here, for *x*<50%, $k\bar{\rho }\;\frac{dC_{p}}{dx}>\bar{\rho }C_{p}\frac{dk}{dx}$, and therefore $\frac{d\alpha }{dx}<0.$ For *x*>50%, however, the non-linear trends in *k* lead to $k\bar{\rho }\;\frac{dC_{p}}{dx}<\bar{\rho }C_{p}\frac{dk}{dx}$ and $\frac{d\alpha }{dx}>0$. As described subsequently, our clinical data suggest that $\frac{d\alpha }{dx}<0$ throughout the range of hydration levels and patients examined.

The values of *ρ*, *C*_p_, and *k* used above are from literature sources and, in some cases, from independent measurements. The densities of dried porcine skin samples (*n*=4, dried in a desiccator for 1 week until no weight change was observed) determined using a graded cylinder of water and an analytical balance are 900±30 kg m^−3^. For skin hydration across a physiological range (approximately 35–50% of secondary water in the outer epidermis^8^), the density increases monotonically with hydration from 900 kg m^−3^ for dry skin to approximately 1020–1150 kg m^−3^ (depending on body location)^38,39^. Measurements by DSC on dried porcine skin yield a dry skin value of *C*_p_ of 1500 J kg ^−1^ K^−1^. The corresponding value for pure water is 4184 J kg ^−1^ K^−1^, and typically reported values for skin are ~3500 J kg ^−1^ K^−1^ (Ref. 40), with a monotonic, linear increase with increased hydration levels. Literature reports suggest that the thermal conductivity, *k*, of dried skin is ~0.2 W m ^−1^ K^−1^ (Ref. 35). The thermal conductivity of water is 0.6 W m ^−1^ K^−1^ (Ref. 41) and typical values for healthy skin are ~0.3–0.5 W m ^−1^ K^−1^ (Refs. 15,^21^). These results also suggest a monotonic increase in *k* with *x*. The non-linear rate of increase illustrated in the computations of Figure 3d is consistent with previous experimental findings^15,21,22,35,42^, but a linear approximation is frequently invoked for the range of clinically relevant hydration levels. For example, in the linear fit shown in Figure 2c (red line), *R*^2^=0.97 and the measured values of *k* are within 3% of linearly fitted values. The values of *k*, *ρ*, and *C*_p_ for dry skin and water listed above define the limits where *x*=0 (perfectly dry skin) and *x*=1 (pure water) in the simulations shown in Figure 3 and Supplementary Figure S6.

### Depth of measurement

**Figure 4:**
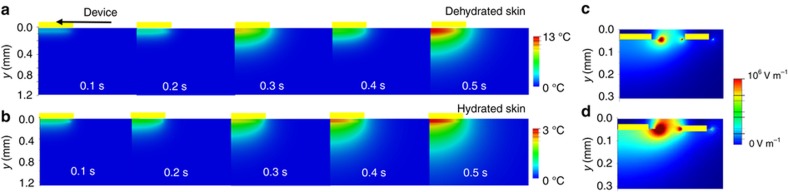
Finite element simulations of heat flow and electrical field distribution in multimodal sensor. (**a**) Time evolution of heat generated by 1-mm diameter circular ETPS element on dehydrated skin (*k*=0.18 W m^−1^ K^−1^, *α*=0.95×10^−7^ m^2^ s^−1^). (**b**) Time evolution of heat from sensor with same geometry as above, on hydrated skin (*k*=0.6 W m-K^−1^, *α*=1.36×10^−7^ m^2^ s^−1^). (**c**) Electric field distribution between concentric impedance sensing elements (200 μm ID, 350 μm OD) supplied with 2 V peak-to-peak voltage, on dehydrated skin (*ε*=1133.6, *σ*=2.04×10^−4^ S m^−1^). (**d**) Electric field simulation of same impedance element as above, on hydrated skin (*ε*=29 010, *σ*=2.93×10^−3^ S m^−1^). ID, inner diameter; OD, outer diameter.

Magnetic resonance imaging studies suggest that changes in epidermal hydration profiles typically associated with topical application of compounds occur to a depth of ~300 μm^43^. FEA shows that the penetration depth of the thermal signal from an individual ETPS element for dehydrated and hydrated skin (Figures 4a and b) is comparable to this value. In these models, the device consists of a heater with radius 0.5 mm (100 nm Au, as in the inset of Figure 1b), encapsulated by two layers of Ecoflex (50 μm at top and 10 μm at bottom, thermal conductivity 0.15 W m^−1^ K^−1^, and thermal diffusivity 0.091 mm^2^ s^−1^ (Ref. 1), air convection coefficient of 10 W m^−2^ K^−1^, total heating power of 3.7 mW), mounted on skin (*k*=0.08 W m K^−1^, *α*=0.045 mm^2^ s^−1^, and 0.6 W m K^−1^ and 0.136 mm^2^ s^−1^ for dehydrated and hydrated skin, respectively^3,4^). The majority of the thermal response occurs in a region between 0 and 400 μm below the surface of the skin, spatially coincident with changes expected from application of lotions. Simply soaking the skin in water results in a different hydration profile^43^, with the majority of the change concentrated in the most superficial layers of skin (<50 μm below skin surface). FEA of an individual EIS element (thickness=100 nm) for dehydrated (permittivity 1133.6, and conductivity 2.04×10^−4^ S m^−1^ (Ref. 8)) and hydrated (permittivity 29010 and conductivity 2.93×10^−3^ S m^−1^ (Ref. 8) skin, with a backing layer of Ecoflex (50 μm, permittivity 2.5 (Ref. 6 and conductivity 1×10–13 S m^−1^ (Ref. 7)), 10 kHz frequency and 2 V potential difference between the concentric electrode elements^20^ appear in Figures 4c and d. Here the electric field concentrates in a region between 0 and 50 μm below the surface of the skin. These findings support the utility of multimodal characterization capabilities. In addition, the measurement depth of both types of sensors changes with their overall dimensions. For an ETPS sensor, increasing the size reduces edge effects and in-plane heat dissipation, thereby facilitating measurements into the depth of the skin. For an EIS element, the measurement depth is equal to roughly half of the spacing between the concentric electrodes^25,44,45^.

### Uncertainty and precision of fitting models

**Figure 5:**
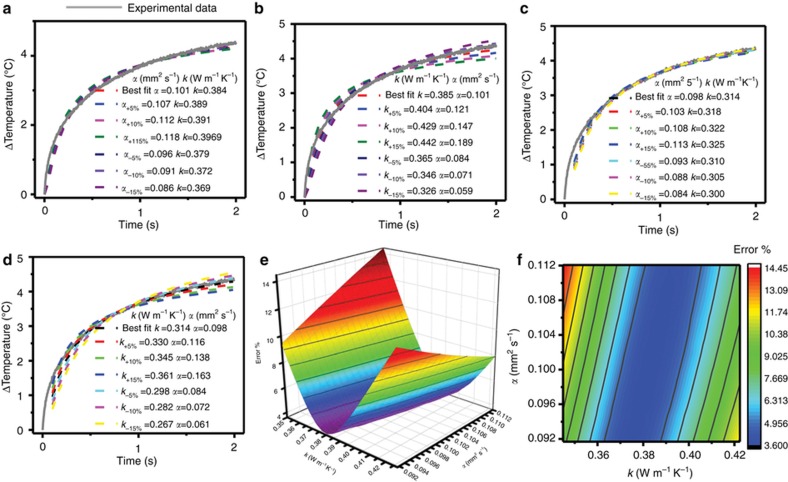
Errors and uncertainties with associated with analytical fitting models. (**a**) Variability in fit induced by changing thermal diffusivity by fixed amounts (±5% ± 10%, ±15%) and recalculating thermal conductivity to its new best-fit value. (**b**) Variability in fit induced by changing thermal conductivity by fixed amounts (±5%, ±10%, ±15%) and recalculating diffusivity to its new best-fit value. (**c**) and (**d**) are calculated using the same procedure as **a** and **b**, respectively, but without fitting the first 5% (0.1 s) of the transient rise curve. (**e**) Error surface computed for the two parameter fits of the entire curve for the data set shown in **a**–**d**. (**f**) Error surface projected onto two-dimensional (2D) map. All experimental data, shown with gray lines in (**a**–**d**) are for porcine skin at 90% saline content (by weight).

In general, the fitting process yields uncertainties in *k* that are significantly lower than those in *α*^24^. Calculation results in Figure 5 reveal the sensitivity of computed results on these two parameters, corresponding to the case of representative data from porcine skin. The study involves systematically increasing/decreasing the best-fit value of *α* (0.10 mm^2^ s^−1^) by ±5%, ±10%, and ±15%, fixing this value and refitting the data while allowing only *k* to change. Increasing *α* by 5% results in a 1% increase in *k*, with similarly small increases in *k* for 10% (1.7% rise in *k*) and 15% (3.2% rise in *k*) increments in *α*. Decreasing *α* by 5%, (1.3% decrease in *k*), 10% (3% decrease in *k*), and 15% (3.9% decrease in *k*) yields similar results. In addition, the quality of the fit does not change significantly for values of *α* in this range, as in Figure 5a. By comparison, the results depend strongly on *k*. Figure 5b shows outcomes from a sensitivity study similar to that described above, but in which *k* varies systematically, and the refitting involves changes only in *α*. Increases of 5, 10, 15% in *k* yield increases of 16, 45 and 87% in *α*, respectively. Similar results are obtained for decreases in *k*. This increased sensitivity to *k* is also apparent from visual inspection of Figure 5b. These functional dependencies can be summarized in the error maps and surfaces in Figures 5e and f. Here the error corresponds to the sum of the squares of the differences between the computed and experimental temperature values for measurements from a representative porcine skin sample. A large number (nearly 1000) of combinations of values of *k* and *α* encompass a range of ±15% from the best-fit values for each parameter. The strongly elliptical shape of the error map with major axes largely aligned with the *α* axis, is consistent with the fitting results described above.

Consistent with the effective medium model, *k* displays a linear relationship with hydration. This dependence, together with the strong sensitivity of the thermal response to *k*, makes it a good choice for determining hydration. Small variations in the transient thermal response, such as those within the range captured in Supplementary Figure S7, result in similar variations in thermal conductivity. Comparison to characteristic features of the transient thermal response curve, such as the temperature at a fixed time interval (in this case, 2 s) after the beginning of the actuation, as shown in Figure 2c, shows variations that are comparable those observed in *k*, suggesting minimum variability introduced by the fitting procedure. By contrast, *α* varies in a non-linear manner with hydration, and small variations in the transient thermal response curve result in much larger variations in computed values of *α*. In this sense, *α* has greater utility in capturing, with *k*, *ρC*_p_ than it does in determining hydration.

Overall, in determining hydration, the thermal and hydration measurement modalities are complementary. Thermal measurements (1) are less susceptible to contact resistances due to the increased penetration depth of the thermal signal (as seen in the FEA shown in Figure 4; (2) do not require direct contact, allowing for a soft elastomeric layer to provide further insulation from leakage currents into the skin (in addition to the stiff, PI layer)^15^, in addition to imparting robustness to the device, unlike impedance-based sensors where the electrodes need be in direct contact with skin^19^; and (3) offer advanced capabilities in temperature^16^ and blood flow mapping^17^ using similar resistive elements. Impedance-based measurements (1) build on an extensive library of knowledge on skin-based electrical measurements^25,44^; (2) provide versatility in probing volumes derived from the ability to use a wide range of applied frequencies^45^; (3) yield physical information about skin through both the capacitive reactance (permittivity) and the resistance (conductivity)^23^ embedded in the measured impedance; and (4) enable comparisons with existing commercial measurements, almost all of which rely on electrical sensing^9^. Both measurements offer comparably short speeds, with a complete impedance spectral sweep requiring roughly the same amount of time as a time-multiplexed measurement of 16 individual ETPS elements (both ~10 s).

### Effects of compounds on patients

**Figure 6:**
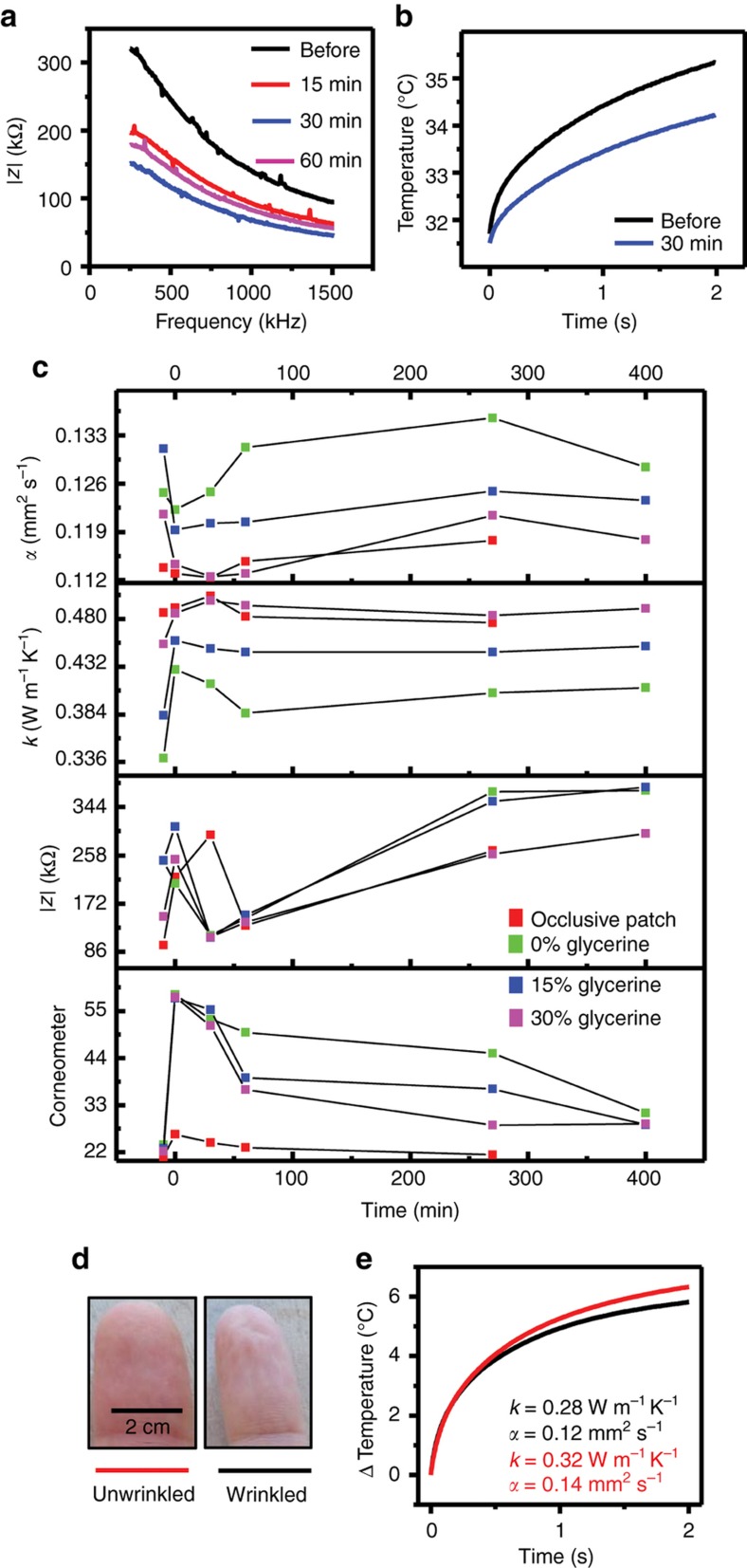
Effect of treatments on epidermal hydration. (**a**) Measured impedance spectra at different time points after application of 15% glycerine compound on volar forearm. (**b**) Measured temperature response at different time points before and after application of 15% glycerine compound on volar forearm. (**c**) Representative thermal diffusivity, conductivity, impedance, and corneometer measurements at different time points for four different treatments on volar forearm of patient. (**d**) Optical image of left index finger before (left) and after (right) placing in warm water for 45 min to induce wrinkling. (**e**) Thermal conductivity and diffusivity measured by ETPS element on finger tip before (red) and after (black) wrinkling.

Representative changes in measured skin properties, demarcated by age and treatment-type appear in Figure 6. The application of the humectant-based cream (15% glycerin, by weight) results in a strong reduction in AC impedance, consistent with the presence of free and bound water, thereby reducing both the permittivity and the resistivity, as shown in Figure 6a. This trend continues until 30 min after the initial compound application, at which time it reaches steady-state. Figure 6b shows the effect of the same compound on the thermal conductivity of skin. The lowered slope of the transient temperature rise curve suggests a lower thermal conductivity 30 min after the application of the compound.

Figure 6c shows the time series evolution of epidermal properties on a representative patient. Application of the compounds and the covering the skin with an occlusive patch for 15 min lead to decreases in AC impedance and increases in thermal conductivity, both consistent with increased hydration level. The corneometer indicates a large initial change in moisture content, followed by a return to a lower value, and then finally to the baseline, pre-treatment level after the removal of the compound. The effects of the occlusive patch occur on comparatively short time scales, with a return to baseline values within 30 min after the initial measurement, due to rapid evaporation of the accumulated transepidermal water.

Thermal transport properties, after registering an initial decrease in thermal conductivity, and decrease in thermal diffusivity remain largely stable for the remainder of the measurement period. These opposing trends in thermal conductivity and diffusivity are consistent with simulations such as those in Figure 3f. The presence of thin, residual films of topically applied compounds can, in certain cases, directly influence the measurement. Such affects are more significant for electrical impedance than for thermal due to the shorter penetration depth of the former compared to the latter.

As another example of measurement utility, Figures 6d and e illustrate the effects of water-induced wrinkling of human skin on thermal transport properties. A healthy patient (female, 31 years old) gently immersed right index finger in warm water for 90 min, with thermal measurements performed before and after. The measured thermal conductivity and diffusivity decrease appreciably, from 0.32 to 0.28 W m ^−1^ K^−1^ and from 0.14 to 0.12 mm^2^ s^−1^, respectively. These data are consistent with recent studies that suggest wetness-induced wrinkling is a neurological response that causes vasoconstriction^46^. Independent measurements of volume change of the finger showed no appreciable change before and after wrinkles were induced.

### Clinical evaluation

Clinical studies demonstrate both the utility of the devices in scaled, rapid measurements across a large cohort and the ability to quantify time-dependent changes in hydration. Topical application of three different compounds with varying glycerin concentrations (0, 15, and 30% respectively) on distinct locations of the volar forearm served as a chemical stimulus to induce these changes. An occlusive patch mounted for 15 min on the skin formed a physical barrier to transepidermal water loss, thereby providing a complementary, physical mechanism. Scatterplot matrices showing individual correlations between the various measured quantities—impedance, thermal conductivity, thermal diffusivity, volumetric heat capacity, and commercially measured hydration and transepidermal water loss (TEWL) appear in Supplementary Figure S2.

**Figure 7:**
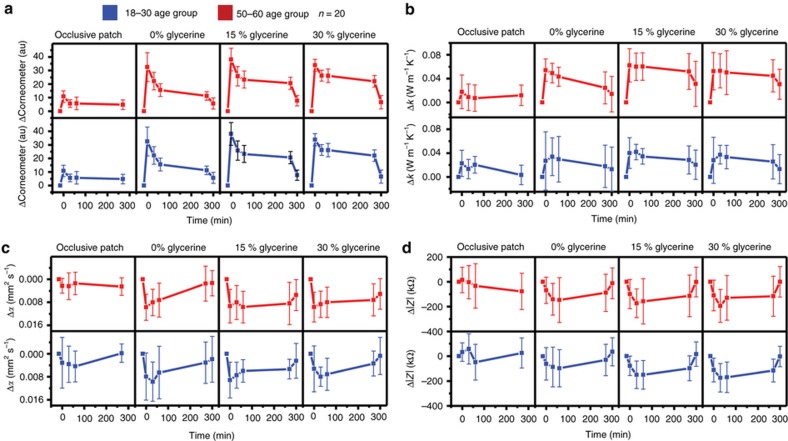
Time series averages of change in baseline due to epidermal treatment, of entire clinical data set (*n*=20), of (**a**) corneometer, (**b**) thermal conductivity, (**c**) thermal diffusivity, and (**d**) AC impedance. The treatments are applied at the time point *t*=0 min. Error bars represent standard deviations.

Time series changes of each measured quantity, organized by age and treatment-type appear in Figure 7. Here the time that defines the baseline, pre-treatment state is set at 15 min prior to application of compounds or occlusive patches (0 min). Error bars represent the standard deviation observed across all 20 patients.

Figure 7a summarizes the results of measurements with a corneometer. For both age groups, the first measurement taken immediately following the removal of the occlusive patch shows an increase in hydration over the baseline, pre-patch value, but with significant variability from patient to patient. The values quickly return to their baseline levels, between 30 and 60 min after the removal of the patch, for both age groups. The application of glycerine-based compounds induces a strong effect on both age groups, starting with an initial increase immediately after application followed by a decrease to a steady-state value, where it remains for 4.5 h (time point 270 min). After the removal of the compound (time point 300 min) the hydration returns approximately to the baseline, pre-treatment values. These observations suggest an important role for a thin, residual layer of the compounds. The measurements do not show a strong dependence on the compound formulation, in contrast to other studies that suggest a clear variation with concentration of glycerine-based compounds^47^.

Time series changes in thermal conductivity appear in Figure 7b. As in the case of the corneometer, the occlusive patch has little effect on measured conductivity. Application of the compounds, by contrast, induces a large and measurable effect on the thermal conductivity. In addition, a strong, qualitative dependence on formulation of the compounds is also apparent. The 0% glycerine induces an initial increase followed by a steady, linear decay. Removal results in a small further decrease, to close to pre-treatment values. For non-zero glycerine concentrations (15 and 30%), however, removal fails to return thermal conductivities to their baseline values, despite a small decrease. The finding suggests an induced physiological change in the skin. The increased thermal conductivity of the skin at 15% concentration is consistent with the present understanding of glycerine as predominantly a humectant at lower concentrations and predominantly an emollient at higher concentrations^47^. Changes in thermal diffusivity with time appear in Figure 7c. The reduction in diffusivity after the treatments can be explained by high specific heat capacity of water, which is trapped in the stratum corneum. As with thermal conductivity, removal of the compound does not result in a return to baseline values, suggesting changes associated with sub-surface layers of skin. The stronger effect on older skin is consistent with the increased diffusivity of older skin due to its diminished diffusion barrier.

Figure 7d presents changes in measured AC impedance with time. As is the case with the other measurement modalities presented here, the effects of the occlusive patch are negligible. The application of the compounds results in a decrease in AC impedance, associated with both a reduced resistance and reactive capacitance due to the presence of free and bound water molecules. The large error bars, the relative invariance of the changes with the composition of the compound and the return to baseline values on removal of the compounds suggest measurements of much more superficial skin layers, largely confined to the stratum corneum, as verified by the FEA shown in Figure 4.

## Conclusions

The multimodal sensors presented here build on existing concepts in epidermal electronics to provide options in real-time, *in vivo* hydration assessment of human skin. Different measurement modalities offer pathways to understanding the distinct physics associated with epidermal thermal and electrical transport, respectively. These concepts are easily extended to sensors with individual elements capable of probing both electrical and thermal properties, depending on the type and duration of power supply. Extensive *in vitro* and *in vivo* tests establish working principles, and viability in a clinical setting. Results on 20 patients and with a variety of stimuli suggest strong agreement with existing state-of-the art tools, paving the way for future embodiments of such devices to be widely used in both clinical and at-home monitoring.
