# Supplementary material for: Multimodal epidermal devices for hydration monitoring
Source: Microsyst Nanoeng. 2017 Jun 5;3:17014. doi: 10.1038/micronano.2017.14 (PMC6444991; doi:10.1038/micronano.2017.14)
Supplement: Supplementary Information [file micronano201714-s1.pdf]

Supplementary file

Multimodal epidermal devices for hydration monitoring

Siddharth Krishnan[1,2,*], Yunzhou Shi[3,*], R. Chad Webb[1], Yinji Ma[4], Philippe Bastien[5], Kaitlyn E. Crawford[1,2], Ao Wang[4], Xue Feng[6], Megan Manco[7], Jonas Kurniawan[1], Edward Tir[1], Yonggang Huang[4], Guive Balooch[3], Rafal M. Pielak[3] and John A. Rogers[8]

Microsystems & Nanoengineering (2017) **3,** 17014; doi:10.1038/micronano.2017.14; Published online: 5 June 2017

SUPPLEMENTAL NOTE S1: FABRICATION OF SENSORS

Fabrication procedure for 1 × thermal/flow sensor array—both epidermal and implantable

Prepare polymer base layers

1. Spin coat with PMMA 495-A2 (poly(methyl methacrylate), spun at 3000 rpm for 45 s). 495-A4 will also work;
2. Anneal at 180 °C for 1 min;
3. Spin coat with polyimide (PI, poly(pyromellitic dianhydride-co-4,4′ -oxydianiline), amic acid solution, Sigma-Aldrich, spun at 6000 rpm for 45 s);
4. Anneal at 110 °C for 30 s;
5. Anneal at 150 °C for 5 min;
6. Anneal at 250 °C under vacuum for 1 h.

Deposit first metallization

1. E-beam 10/100 nm Cr/Au Pattern photoresist (PR; Clariant AZ5214, 3000 rpm, 30 s; Bake 110 °C, 1 min) with 365 nm optical lithography through iron oxide or Cr Mask (Karl Suss MJB3 or MJB4) for 6 s. Cr is for Adhesion, Au is the main sensor layer;
2. Develop in aqueous base developer (MIF 917);
3. Etch Cr with Cr Etchant;
4. Etch Au with Au TFA Etchant (KI- KOH solution);
5. Remove PR w/ Acetone. Rinse thoroughly with water (2 cycles).

Deposit second metallization

1. E-beam 20/500/20/25 nm Ti/Cu/Ti/Au;
2. Pattern photoresist (PR; Clariant AZ5214, 3000 rpm, 30 s; Bake 110 °C, 1 min) with 365 nm optical lithography through iron oxide mask (Karl Suss MJB3 or MJB4). Expose for 6 s;
3. Develop in aqueous base developer (MIF 917);
4. Etch Au with Au TFA etchant;
5. Etch Ti w/ BOE;
6. Etch Cu w/ CE-100;
7. Etch Ti w/ BOE;
8. Remove PR w/ Acetone, IPA.

Apply encapsulation

1. Spin coat with polyimide (PI, poly(pyromellitic dianhydride-co-4,4′ -oxydianiline), amic acid solution, Sigma-Aldrich, spun at 6000 rpm for 45 s);
2. Anneal at 110 °C for 30 s;
3. Anneal at 150 °C for 5 min;
4. Spin second coat with polyimide (PI, poly(pyromellitic dianhydride-co-4,4′ -oxydianiline), amic acid solution, Sigma-Aldrich, spun at 3000 rpm for 45 s);
5. Anneal at 110 °C for 30 s;
6. Anneal at 150 °C for 5 min;
7. Anneal at 250 °C under vacuum for 1 h, in designated PI oven;
8. Pattern photoresist (PR; Clariant AZ4620, 3000 rpm, 30 s; Bake 110 °C, 3 min) with 365 nm optical lithography through iron oxide mask (Karl Suss MJB3). Expose for 15 s;
9. Develop in aqueous base developer (AZ 400 K, diluted 3:1);
10. Etch in March RIE (200 mTorr, 150 W, 20 sccm O_2, ~ 1800 s). If using nanomaster, use 200 W Polymer recipe for 20 min.

Release, pick up and print onto Silicone Elastomer

1. Release in Acetone at 50C for 1–5 min (have to observe closely-lots of variability in release time);
2. Pick up with 3 M Water Soluble PVA Tape;
3. E-Beam Deposit 3/30 nm Ti/SiO_2 on Device Side of Tape;
4. UV-O treat PDMS (either ecoflex or Sylgard 184 10:1) for 5 min;
5. Stick Ti/SiO_2 side of device onto UV-O treated side of PDMS;
6. Dissolve PVA tape in DI Water on hot plate at 100 °C.

SUPPLEMENTAL NOTE S2: TRANSIENT PLANE SOURCE (HOT DISC) ALGORITHM

A major limiting factor preventing the complete deployment of the transient plane source formulation is the computationally expensive nature of the algorithm. In principle, for each individual time point, the quantity τ is computed, from which $D(\tau)$ can be obtained. A standard fitting algorithm requires this quantity to be computed for each experimental time point, and then iteratively until a local minimum in the error function is obtained. To significantly simplify this procedure, we first computed the quantity $D(\tau)$ for the entire range of experimentally observed values of τ. This creates a 50 000 point mesh for $0 < \tau < 15$. This computed curve appears in Supplementary Figure S5a. This high-resolution mesh implies a one-time computational cost, but the resulting tabulated values can be easily stored in digital memory after this initial computation. In implementing the curve-fitting algorithm, the quantity $D(\tau)$ does not need to be computed for each time point, but can simply be looked up (and rounded up to the nearest corresponding computed value). On a standard i7

[1]Department of Materials Science and Engineering, Frederick Seitz Materials Research Laboratory, University of Illinois at Urbana-Champaign, Urbana, IL 61801, USA; [2]Department of Materials Science and Engineering, Northwestern University, Evanston, IL 60208, USA; [3]L'Oreal Tech Incubator, California Research Center, 953 Indiana Street, San Francisco, CA 94107, USA; [4]Department of Civil and Environmental Engineering, Mechanical Engineering, Materials Science and Engineering, Northwestern University, Evanston, IL 60208, USA; [5]L'Oréal Research and Innovation, 1 Avenue Eugène Schuller, Aulnay sous Bois 93601, France; [6]Department of Engineering Mechanics, Center for Mechanics and Materials, Tsinghua University, Beijing 100084, China; [7]L'Oréal Early Clinical, 133 Terminal Avenue, Clark, NJ 07066, USA and [8]Departments of Materials Science and Engineering, Biomedical Engineering, Chemistry, Mechanical Engineering, Electrical Engineering and Computer Science, and Neurological Surgery; Center for Bio-Integrated Electronics; Simpson Querrey Institute for Nano/biotechnology; Northwestern University, Evanston, IL 60208, USA
Correspondence: Rafal M. Pielak (RPIELAK@rd.us.loreal.com) or John A. Rogers (jrogers@northwestern.edu)
*These authors contributed equally to this work

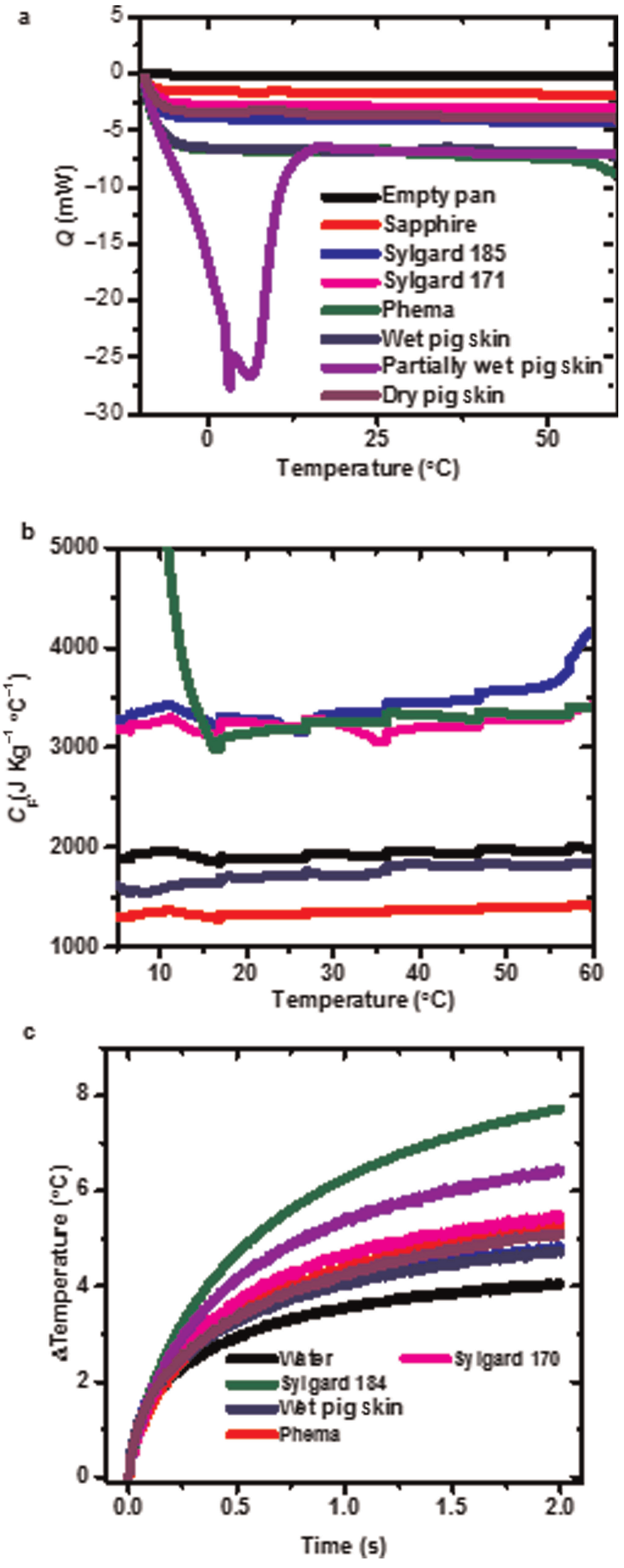

Figure S1 DSC Scans and transient temperature response on test materials. (**a**) Temperature as a function of applied power for the test materials listed, for a heating rate of 5 ° C min^{-1}. (**b**) Calculated specific heat capacity across temperature range. (**c**) Transient temperature rise curve using ETPS for the same test materials.

processor running Windows 7 OS, this reduced the computation time from 4 days to <10 s for each data point. A resulting curve fit on a representative porcine skin sample is shown in Supplementary Figure S5b.

SUPPLEMENTAL NOTE S3: SIMPLE, ANALYTICAL EFFECTIVE MEDIUM MODELS FOR PREDICTING TRENDS IN THERMAL CONDUCTIVITY WITH HYDRATION

The thermal conductivity of a skin-water composite, $k_{composite}$, can also be modeled by a simple rule of mixtures, as was done for the density and specific heat capacity in equations (9 and 10). The dependence of $k_{composite}$ on water content, x, $(0<x<1)$ can be captured using two different models that represent upper and lower bounds. The first, a parallel model, appears schematically in Supplementary Figure S6a. Here, the temperatures at the boundaries of the matrix define by $y=0$ and $y=d$ are fixed at $T=0$ K and $T=1$ K respectively, with the top and bottom boundaries assumed to be adiabatic. An increase in water content, x, corresponds to an increase in the thickness of the water layer in this matrix. Pure water is more thermally conductive than dry skin ($k_{water}=0.6$ W m^{-1} K^{-1}, $k_{dry\ skin}=0.2$ W m^{-1} K^{-1}), and the thicker water layer creates lower resistance to heat flow, and corresponds to a higher value of $k_{composite}$. The thermal conductivity of this system, $k_{composite,\ parallel}$ can be written down mathematically as:

$$k_{composite,\ parallel} = x k_{water} + (1 \quad x) k_{dry\ skin} \qquad (S3.1)$$

A different model, assuming a skin-water composite constructed such that the skin and water are in series, appears schematically in Supplementary Figure S6b. As with the parallel model, the temperature as at the boundaries defined by $y=0$ and $y=d$ represent are kept fixed at $T=0$ K and $T=1$ K respectively, and the top and bottom boundaries are assumed to be adiabatic. In this system, $k_{composite,\ series}$ can be modeled as a function of x using an inverse rule of mixtures and can be written down as:

$$k_{composite,\ series} = \frac{x}{k_{water}} + \frac{(1 \quad x)}{k_{dry\ skin}}^{\ 1} \qquad (S3.2)$$

An exact, theoretical formulation for the 2D case shown in Figure 3a can be written down as[36]

$$k_{composite,\ 2D} = k_{dry\ skin}\left[\frac{(p+1)+x(p \quad 1)}{(p+1) \quad x(p \quad 1)}\right] \qquad (S3.3)$$

where $p = k_{water}/k_{dry\ skin}$. Similarly, for the 3D case, $k_{composite}$ can be written down as:

$$k_{composite,\ 3D} = k_{dry\ skin}\frac{(p+2)+2x(p \quad 1)}{(p+2) \quad x(p \quad 1)} \qquad (S3.4)$$

Theoretical curves corresponding to Supplementary Equations (S3.1)–(S3.4), appear in Supplementary Figure S6c. The parallel model (black curve) is linear, and defines an upper bound, while the series model (red curve) is nonlinear and defines a lower bound. This linearity in the case of the parallel model and nonlinearity in the case of the series model are consistent with established theory on the rule of mixtures. The FEA-computed curves, as described in section ``RESULTS AND DISCUSSION'', also appears in Supplementary Figure S6c (blue and pink points), and closely match the theoretical curves (blue and pink lines) corresponding to Supplementary Equations (S3.3) and (S3.4).

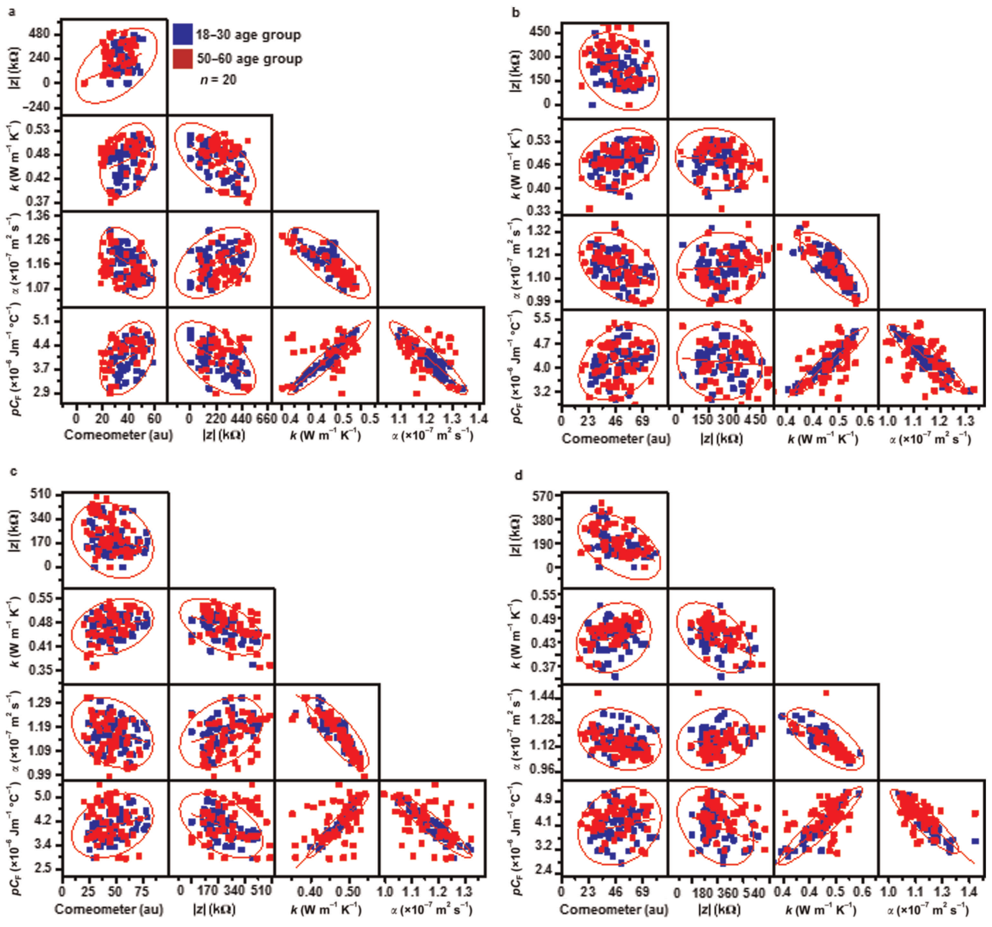

Figure S2 Correlations for clinical data: Boxplot matrices correlating measured and derived parameters for 18–30 (blue) and 50–60 (red) age groups when applied treatment is: (**a**) occlusive patch, (**b**) 0% glycerine compound, (**c**) 15% glycerine compound, (**d**) 30% glycerine compound.

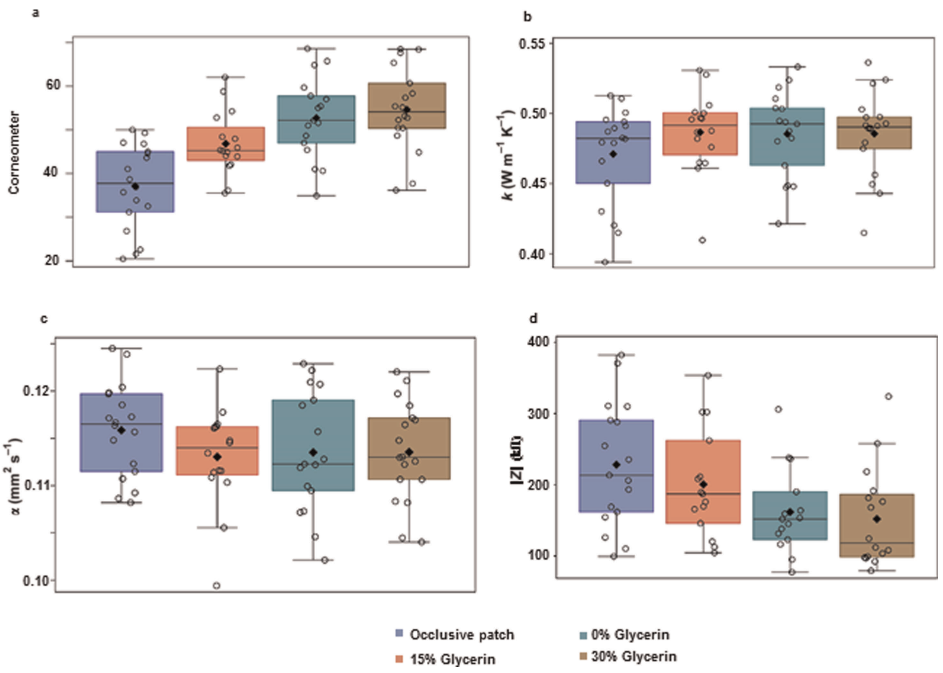

Figure S3 Boxplot representation for clinical data for (**a**) corneometer, (**b**) thermal conductivity from ETPS sensors, (**c**) thermal diffusivity from ETPS sensors, and (**d**) impedance from EIS.

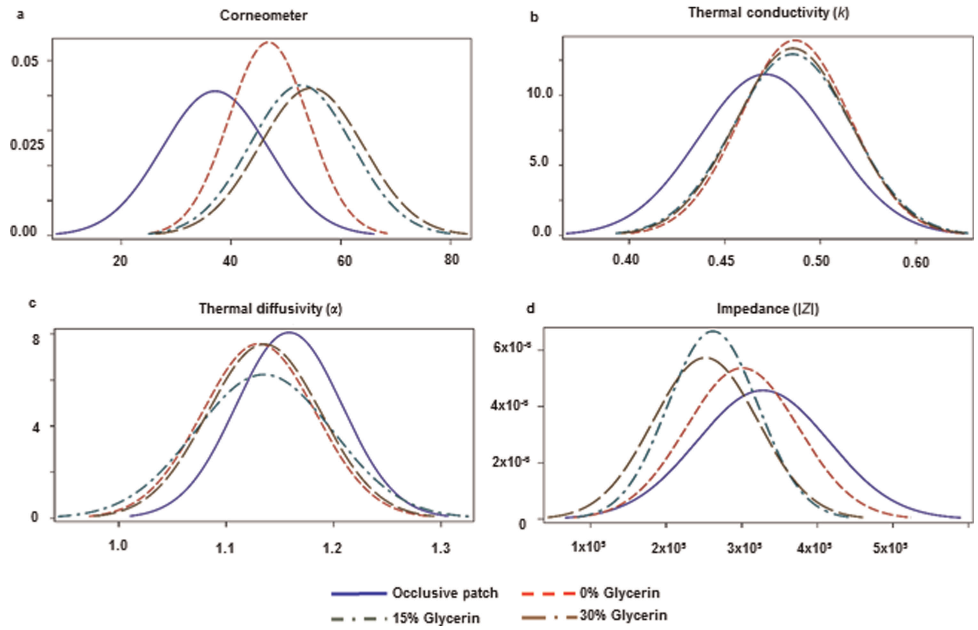

Figure S4 Area-under-curve (AUC) analysis for clinical data for (**a**) corneometer, (**b**) thermal conductivity from ETPS sensors, (**c**) thermal diffusivity from ETPS sensors, and (**d**) impedance from EIS.

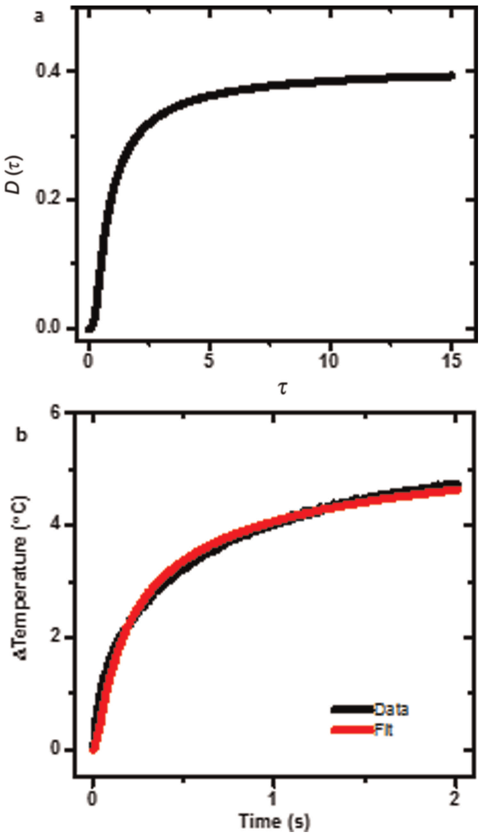

Figure S5 Numerical curve-fitting algorithm. (**a**) Non-dimensional parameter $D(\tau)$ computed for range of τ encountered in experiments. This pre-computed curve is used to repeatedly curve fit the data, thereby significantly reducing the computation time. (**b**) Representative data set and fit on human skin.

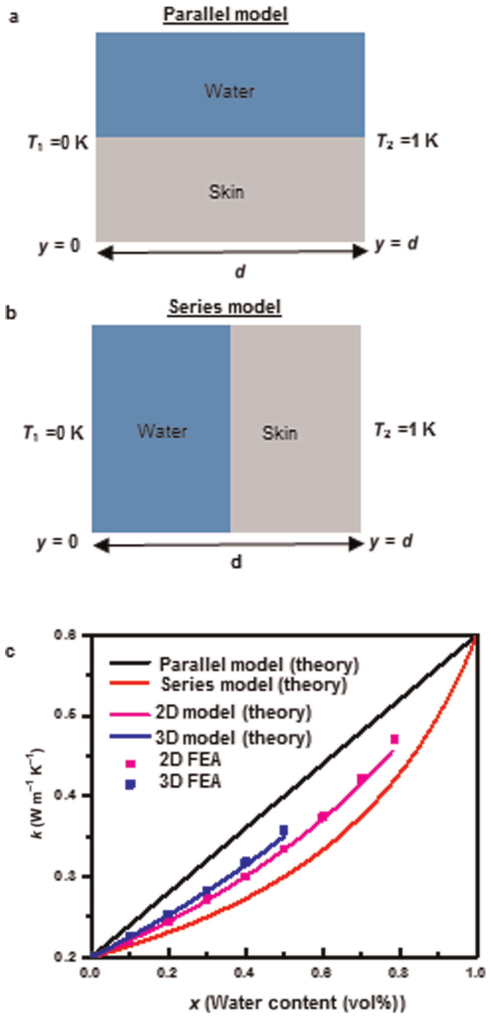

Figure S6 Effective medium modeling for water-skin composite. (**a**) Schematic illustration of parallel model of skin-water composite. (**b**) Schematic illustration of series-model for skin-water composite. (**c**) Thermal conductivity variation with water content, *x*, $(0 < x < 1)$ calculated using parallel and series model (red and black curves) and using FEA (blue curve).

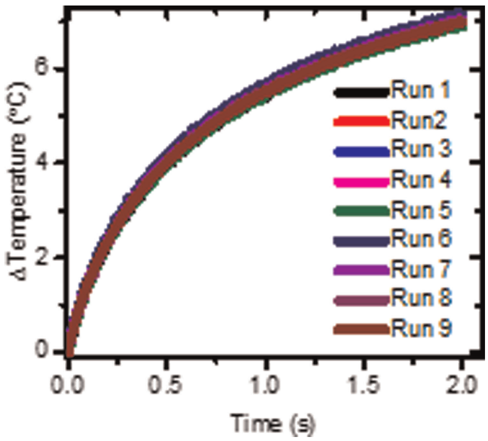

Figure S7 Reproducibility: 9 successive measurements made on test material (PDMS, sylgard 184, 10:1), by removing and re-applying epidermal sensor each time.
